# Millets: A Nutritional Powerhouse With Anti-cancer Potential

**DOI:** 10.7759/cureus.47769

**Published:** 2023-10-26

**Authors:** Mansha Gupta, Dina Medhanie Asfaha, Govintharaj Ponnaiah

**Affiliations:** 1 Medicine, Kasturba Medical College, Manipal, Manipal, IND; 2 Medicine, Orotta School of Medicine, Orotta Hospital, Asmara, ERI; 3 Molecular Biology/Plant Breeding and Genetics, International Crops Research Institute for the Semi-Arid Tropics (ICRISAT), Hyderabad, IND

**Keywords:** micronutrients, bioactive compounds, anti-cancer properties, nutrition, millets

## Abstract

Millets are important food crops widely grown by smallholder farmers in the arid and semi-arid regions of the world. Millets are rich in protein, dietary fiber, micronutrients, and have a low glycemic index (GI) and desirable bioactive compounds. Due to their higher nutritional content, millets are popularly known as "nutricereals". Coinciding with the United Nations and the Food and Agriculture Organization's declaration of 2023 as the "International Year of Millets," this review underscores the nutritional value of these grains from the Poaceae family.

The consumption of nutricereals is associated with several health benefits including lowering of blood sugar levels (diabetes), controlling blood pressure, and providing protection against thyroid, cardiovascular, and cancer diseases. A review of the literature from PubMed and Google Scholar was done focusing on the health benefits and anti-cancer properties of different millets. Millets have a rich content of macronutrients like carbohydrates and proteins, as well as micronutrients and bioactive compounds, including dietary fibers, essential fatty acids, and phytochemicals. This article explores millets' nutritional elements, i.e., macronutrients, micronutrients, and bioactive compounds, and provides insights into the types of carbohydrates present, the prebiotic function of dietary fibers, and millets' low GI. The study identified the mechanisms by which millets may deter cancer growth, focusing on the roles of dietary fibers, plant protease inhibitors, and bioactive peptides. Additionally, it compared the mineral and vitamin content of millets to other common grains, such as rice and wheat, and explored the potential health advantages of millets over other cereal crops. This review systematically investigated the health advantages of millets, particularly, their anti-cancer capabilities. Dietary fibers, plant protease inhibitors, and bioactive peptides present in millets have the capacity to induce apoptosis, inhibit cell proliferation, and interact with gut microbiota leading to potential anti-cancer effects. This review also identified existing challenges in the bioavailability and effective delivery of millets' bioactive peptides, advocating for further research to maximize their health benefits.

## Introduction and background

Food and Agriculture Organization and the United Nations have recognized 2023 as the “International Year of Millets" (IYM 2023) to spread awareness about the health and nutritional benefits of millets. Millets are a group of small-seeded nutrient-dense food crops that are widely grown on marginal lands in dry areas around the world. This group of crops belongs to the family Poaceae and consists of several species including pearl millet (*Pennisetum glaucum*), foxtail millet (*Setaria italica*), proso millet (*Panicum miliaceum*), finger millet (*Eleusine coracana*), kodo millet (*Paspalum setaceum*), little millet (*Panicum sumatrense*), and barnyard millet (*Echinochloa utilis*) [1]. Millets are designated as "nutricereals" as they are rich in macro- and micronutrients, carbohydrates, proteins, dietary fibers, important fatty acids, and a vast number of minerals and vitamins. Millet carbohydrates have less starch compared to the other staple cereal crops. Millet dietary fibers act as prebiotics that help in the development of healthy gut microbiota [2]. The dietary fiber content of millets also slows down the glucose absorption in the small intestine and can help in blood glucose regulation and reducing the glycemic index (GI) of food [3]. Millet proteins are rich in all essential amino acids and are particularly high in cysteine and methionine (sulfur-containing amino acids). Millets are gluten-free cereal grains, and hence can be consumed regularly by celiac patients [4]. The presence of higher levels of phytochemicals such as phenolics, tannins, carotenoids, and flavonoids exhibit strong antioxidant activity and reduce tumor growth [5]. Millets are also well recognized for their potential health benefits, including fighting against many diseases like diabetes, cardiovascular disease, high blood pressure, thyroid disorders, and celiac disease [6]. This review highlights the anti-cancer properties of millets to sensitize scientists to work in this direction.

## Review

Millets: the powerhouse of nutrients

Millets are nutritionally comparable to or superior to major cereal grains. They possess many additional benefits such as high dietary fiber content, gluten free, lower glycemic index, and the availability of abundant bioactive compounds, making them “ideal foods” for human health [7]. The proximate composition and calorific values, dietary fibers, essential amino acids, and micronutrients of millets are provided in Table 1. Millet carbohydrates can be categorized into two: (i) non-structural (sugars, starch, and fructosans) carbohydrates and (ii) structural (cellulose, hemicelluloses, and pectin substances) carbohydrates [8]. The carbohydrate content of millets varies from 56.88 to 72.97 g/100 g. The highest content of carbohydrates has been reported in finger millet, whereas the lowest carbohydrate content is reported in barnyard millet [9]. Millets' dietary and crude fiber content is much higher than that of the other staple cereals like rice, wheat, and maize [4]. The soluble dietary fiber offers many health benefits, preventing constipation, enhancing gut health, and reducing heart disease. Also, millets' dietary fiber reduces the GI of the food. The GI of millets varies from 33 to 65, which is significantly lower than the GI of rice and corn. Furthermore, dietary fiber directly interacts with gut microbiota and releases short-chain fatty acids (SCFAs) through fermentation, which can help prevent pathogen invasion and colonization by reducing the gut pH [10]. Such dietary fibers are known as “prebiotics”. These suppress the growth of pathogens and remove carcinogens from the body and enhance nutrient absorption and immunity [11].

**Table 1:** Millets' nutrient composition ^†^Glycemic Index Scale: low, 55 or less; mid, 56-69; high, 70+ (source: Glycemic Index Foundation, https://www.gisymbol.com/) Source: [4,8]

Crop Name	Proximate and calorific values				Dietary fiber contents				Micronutrient composition										
	Carbohydrates (%)	Protein (%)	Fat (%)	Calorific value (calories/100g)	Total dietary fiber (%)	Insoluble dietary fiber (%)	Soluble fiber (%)	Glycemic index^† ^ (numbers)	Calcium (mg/100 g)	Phosphorus (mg/100 g)	Zinc (mg/100 g)	Iron (mg/100 g)	Thiamine-B1 (mg/100 g)	Riboflavin-B2 (mg/100 g)	Niacin-B3 (mg/100 g)	Folic acid (μg/100 g)	Total carotenoids (μg/100 g)	Vitamin E (mg/100 g)
Pearl millet	67-70.4	10.6-12.6	4.8-5.0	363-412	8.0-13.5	5.0-8.0	2.0-3.0	55.0	27.0-43.0	339	3.1	6.0-17.0	0.3-0.4	0.09-4.0	1.0-3.0	45.5		1
Finger millet	66.5-75.0	6.0-8.2	1.3-1.5	332-376	15.0-20.0	3.6-19.7	2.5-3.0	55.0-65.0	335.0-352.0	250	32.0-41.0	4.0-6.0	0.3-0.5	0.02-0.19	0.8-2.0	18.3	45-199	4.1
Proso millet	62.3-72.2	10.6-12.5	1.8-4.0	341-364	8.5-14.2	7.0-12.0	1.5-2.0	50.2-64.7	20.0-42.0	300	60.6	3.0-5.0	0.6	0.05-0.16	0.5-3.2	15.0	173	1.2
Foxtail millet	61.6-72.4	9.9-12.3	2.5-4.0	351-353	19.1	7.0-17.8	1.3	33.0	7.0-13.5	200-206	2.4-3.7	2.2-4.8	0.4	0.28	4.5		366	3.6
Barnyard millet	49.0-65.5	8.9-11.9	2.2-4.5	300-310	13.6	4.0-14.7	4.2	41.7-50.0	12.0-25.0	280	55.0-60.0	15.0-19.0	0.3	0.1	4.2			
Kodo millet	56.1-74.0	8.3-11.6	1.3-4.2	346-353	6.8-37.8	4.0-6.0	2.0-3.0	49.5	27.0-37.0	300	30.0-35.0	1.8-4.0	0.2	0.09	0.1-1.2	23.1		0.5
Little millet	64.2-67.0	7.6-10.0	2.4-2.8	325-336	6.4-12.2	5.0-7.0	2.0-3.0	41.5-61.8	15.0-17.0	220	32.2-35.1	1.2-1.7	0.3	0.05-0.09	1.3-3.2	9.0	78	1.3
Rice (brown rice)	72.8-75.8	8.5-9.90	1.1-1.3	325-340	0.7-6.0	0.6-4.3	0.7-1.0	65.0-81.0										
Wheat	61.2-66.8	10.0-12.0	1.4-15	310-334	2.8-12.1	2.1	1.0-2.0	44.0-60.0										
Maize	63.2-66.4	8.0-9.5	3.6-4.1	328-338	3.9-13.4	2.3-12.4	0.8-1.2	78.5-86.3										

Millets are reservoirs of many minerals and vitamins. Among millets, finger millet is rich in calcium (348 mg/100 g) [4], which is about eight times higher than wheat. Furthermore, Singh and Raghuvanshi reported that the calcium content estimated on the 36 finger millet genotypes varied from 162 to 487 mg/100 g [12]. Finger millet consumption of 100 g can provide roughly half of the recommended dietary allowance (RDA) for calcium content [13]. The phosphorus content (200-339 mg/100 g) varies widely among millets. According to the recommendation of the Indian Council of Medical Research (ICMR), consumption of millets can supply about 50% of the phosphorus requirement [14]. The zinc content of finger millet, kodo millet, and little millet is found higher than the RDA of 12 mg. The consumption of 100 g of barnyard millet can meet 100% of the RDA for iron content.

Millets also have high amounts of vitamins, especially vitamin B complex and vitamin E. Among millets, proso millet has a higher amount of thiamine, riboflavin, niacin, and total carotenoids whereas highest tocopherol content is found in finger millet. The riboflavin content of millets is several times higher compared to other staple cereals. Pearl millet contains a considerable amount of vitamin E (2 mg/100 g; fat-soluble component) and vitamin A [15]. Vitamins (water-soluble vitamins) play an important role in energy production, while carotenoids and vitamin E serve as antioxidants that support immunity, prevent eye defects, and enhance anti-cancerous properties of the body [16].

Phenolic compounds such as phenolic acids, flavonoids, and tannins are commonly present in millets and play a significant role in the immune system [17]. Millets have more phenols than staple cereals. The presence of ferulic and p-coumaric acids in whole pearl millet grains has the capacity to reduce tumor cells [18]. Among millets, finger millet has the highest phenolic compound content. The major phenolic compounds present in proso millet, finger millet, and foxtail millet are hydroxycinnamic acids. The phenolic compounds extracted from finger millet seed coat slow down glucose absorption, help in ameliorating postprandial hyperglycemia, and partially inhibit pancreatic amylase and alpha-glucosidase activity in carbohydrate metabolism [19]. Flavonoids are more common in free form. Brown-colored finger millet genotypes/cultivars have the highest concentrations of flavonoids and tannins [12]. Millets contain a variety of flavonoids among which luteolin, quercetin, catechin, naringenin, dizein, apigenin, and kaempferol are the most prevalent [18,19]. Regular consumption of millets containing high amounts of flavonoids and tannins lowers the risk of developing cancer, diabetes, cardiovascular disease, and neurological illnesses.

Millets' cancer-fighting compounds and their anti-cancer property

Millets are a source of several types of compounds that have anti-cancerous properties; they are discussed next.

Dietary Fibers

Dietary fiber is an important component present in plant cell walls. Dietary fibers are cell wall polysaccharides and play a significant role in the human gut, helping in digestion and improving gut health. Millet dietary fibers are classified into two, namely, (i) soluble dietary fiber (SDF) and (ii) crude fiber or insoluble dietary fiber (IDF) [20]. SDF is soluble in water and composed of pectins, glucans and some hemicellulose whereas IDF is insoluble in water and contains cellulose, hemicellulose, and lignin. In comparison with IDF, SDF has a higher nutritional content, lowers cholesterol (fatty substances trapped in the GI tract) level, absorbs more water, forms a gel-like structure, ferments the gut bacteria in the large intestine, regulates the immune system, and has anti-tumor properties [21]. The formation of resistant starch (RS) contributes the dietary fiber content that ultimately provides significant health benefits [22]. RS is a functional fiber fraction that plays a significant role as it escapes the enzymatic digestion in the intestinal tract leading to several health benefits [23]. It has been shown that the production of butyrate metabolites from gut microbial fermentation helps stabilize colorectal cell proliferation [24].

Dietary fibers gained more attention as potential therapeutic agents for manipulating gut microbiota and alleviating GI tract inflammation. For example, a recent study found that foxtail millet SDF inhibited the colony formation ability of HCT116 and HT-29 cells and could significantly induce the increase of reactive oxygen species (ROS) and apoptosis of HCT116 and HT-29 cells [25]. In another study, Zhang et al. compared the effects of dietary supplementation of foxtail millet and rice on colorectal cancer; it was found that foxtail millet inhibited the phosphorylation of STAT3 and the related signaling pathway proteins involved in cell proliferation, survival and angiogenesis (mediated by the activation of gut receptors, i.e., aryl hydrocarbon receptor, or AHR, and G-protein-coupled receptors, or GPCRs). Furthermore, millet treatment increased the abundance of *Bifidobacterium *and *Bacteriodales_S24-7*,* *when compared to the rice-treated mice [26].

Plant Protease Inhibitors

Plant protease inhibitors are multifunctional proteins. Proteases are involved in a variety of biological processes, such as inflammation, infection, extracellular matrix breakdown, blood coagulation, programmed cell death, tumor invasion and metastasis [27,28]. Protease inhibitors are classified into six groups: cysteine, serine, threonine, glutamic acid, aspartate proteases, as well as matrix metalloproteinases, based on the type of amino acid present in the active site of the protease and the mechanism of peptide bond cleavage. Numerous studies have been conducted on protease inhibitors; the plant protease inhibitors are classified into families such as Bowman-Birk, Kunitz, Potato I, Potato II, Serpine, Cereal, Rapeseed, Mustard, and Squash [29,30]. These families are distinct from one another in terms of their mass, cysteine concentration, and the number of reactive sites [31].

Among millets, a protease inhibitor is isolated and well characterized in *ragi* (finger millet, *Eleusine coracana*), which is popularly known as “ragi bifunctional inhibitor (RBI)”. RBI is a 14 kDa bifunctional inhibitor purified from ragi seeds [32], is a member of cereal trypsin/α amylase inhibitor family that inhibits α-amylase and trypsin forming a ternary complex simultaneously [33]. It consists of single polypeptide chain containing 122 amino acids including five intramolecular disulfide bonds [34]. A study was conducted by Sen and Dutta to evaluate the anti-carcinogenic activity of RBI in K562 human chronic myeloid leukemia cells [32]. The purified RBI from finger millet seeds suppressed the proliferation and induced apoptosis of K562. The cytotoxicity test showed that the RBI has antiproliferative potential against K562 chronic myeloid leukemia cells, but not against normal human cells.

Bioactive Peptides

In the recent decades, an increasing number of lifestyle-related non-communicable diseases have been found to be heavily associated with incorrect eating habits and sedentary lifestyle; therefore, there is a huge surge in demands of foods with additional health benefits. Foods that provide additional health benefits along with basic nutrition (vitamins, minerals, fiber, protein, or peptides) are known as functional foods [35]. Among these nutritional components, peptides have received significant attention due to extraordinary biological functions and health-related benefits to combat lifestyle-related degenerative diseases. Peptides are a small string of proteins consisting of 2-20 amino acid subunits (encrypted in the parent protein) linked by peptide bonds. Peptides are characterized by low molecular weight (MW, <3 kDa) compounds and become more active when released during enzymatic proteolysis of proteins and during food processing such as cooking, fermentation, and ripening [36,37]. Bioactive peptides are obtained from protein-rich plants and animals. Recently, protein-rich millets have been identified as a novel source of bioactive peptides. For instance, finger millet, barnyard millet, and proso millet are rich sources of antimicrobial proteins/peptides [38]; finger millet and pearl millet show antioxidant activity and foxtail millet peptides possess anti-hypertensive properties [39,40]. In addition to this, millets' bioactive peptides possess several physiological and biological functions that include antioxidant, antimicrobial, anti-hypertensive, immune modulatory, anti-inflammatory, antifungal, antiviral and anti-cancer effects [41,42].

The phytochemicals derived from millets have a variety of biological functions like anti-cancer, antimicrobial and anti-inflammatory activities, suggesting their use as a functional food in the prevention and therapy of diseases. A peroxidase of class III protein extracted from foxtail millet bran, known as “FMBP”, was found to have anti-colon-cancer activity in mice in both in vitro and in vivo studies [43]. The FMBP suppressed colon cancer cell growth by arresting the G1 phase, but apparently had no effect on the normal colon epithelial cells. In the same study, the epithelial-mesenchymal transition (EMT), and FMBP reduced the phosphorylation of JAK1 and its downstream signaling molecular STAT3, followed by the reduced expression of c-Myc and Snail1 of EMT. The STAT3 overexpression could partially reverse the migration inhibition caused by FMBP [44]. Also, the anti-cancer effects of FMBP were mainly achieved through the accumulation of more ROS in colon cancer cells than normal cells, attributed to the down-regulation of NF-E2-related factor 2 (Nrf2) expression, and the reduction of catalase activities and glutathione contents [45]. 

In a study by Kuruburu et al., phytochemical-rich fractions (phenolics and flavonoids) were extracted from foxtail millet seeds using 70% ethanol (FTM-FP) and 10% alkali (FTM-BP). Both fractions showed antiproliferative properties against breast cancer cell lines by inducing cell cycle arrest in the G2/M phase followed by increased DNA fragmentation leading to the accumulation of more cells in the Sub-G1 phase [46]. Also, Zhang and Liu reported that the extracted phenolic and carotenoids from foxtail millet cultivars Jingu28 and Jingu34 inhibited the growth of human breast and liver cancer cells in culture [47].

The antioxidants obtained from both kodo and pearl millet bran extracts exhibited higher anti-proliferative/anti-cancer activities compared with those of the dehulled extracts (Table 2) [48]. The same study also concluded that a low concentration (0.1 mg/ml) of phenolic extracts obtained from the dehulled pearl millet grain showed 1.3 times higher anti-carcinogenic activity than a high concentration (0.5 mg/ml). In addition, it was noted that phenolic compounds isolated from whole pearl millet grain (52.7%) and dehulled grain (43.8%) exhibited greater inhibitory effects against the colon cancer cell line HT-29 in comparison to their kodo millet counterparts. Vanillin (4-hydroxy-3-methoxybenzaldehyde) extracted from proso and barnyard millets showed that the proso millet extract suppressed the cellular proliferation of HT-29 cells significantly when treated with 250 µg/ml and 1000 µg/ml concentrations of phenolic extracts followed by a 48-h incubation period, while the barnyard millet extract moderately inhibited the proliferation of the HT-29 cell line at the same concentrations of phenolic extracts and within the same time period [49]. The same extracts applied to the MCF-7 cell line at the same concentrations and for the same time period showed that both the extracts had significantly inhibited the proliferation of MCF-7 cells through G0/G1 phase cell arrest and increased the apoptotic cells in the sub-G0 phase in a dose-dependent manner [50].

**Table 2:** Millets' bioactive peptides and their effects on cancer cell lines TPC, total phenolic content; BPIS, bound polyphenol of inner shell; SFA, saturated fatty acids; MUFA, monounsaturated fatty acids

S. no.	Crop	Functional factor	Cancer		Mechanism of action	References
			Type	Cell line		
1	Pearl millet	TPC, hydroxyl and peroxyl radical inhibition	Colon cancer	HT-29	Millet grain phenolic extracts suppressed the cancerous cell growth, which was majorly time and dose dependent. At a higher concentration (0.5 mg/ml), hulled pearl phenolic extracts exhibited 68% of cellular proliferation inhibition activity. On the other hand, at a lower concentration (0.1 mg/ml), the whole and dehulled pearl millet grain phenolic extracts had a higher inhibition activity of 53% and 44%, respectively, against HT-29 cells, compared to kodo millet counterparts.	[48]
2	Foxtail millet	35 kDa protein FMBP extracted from foxtail millet bran	Colon cancer	DLD1, SW480, HT-29 and human colon epithelial cell line FHC	FMBP, homologous to peroxidase enzyme, suppressed colon cancer cell growth through induction of G1 phase arrest. The phenolic extract induced caspase-dependent apoptosis in colon cancer cells leading to the loss of mitochondrial trans-membrane.	[43]
		BPIS from foxtail millet bran (vitexin and syringic acid)	Breast cancer	MDA-MB-231 and MCF-7	BPIS suppressed the breast cancerous cells through blocking the conversion of SFA to MUFA. BPIS also reduced the GRP78 protein that inhibits the expression of SREBP-1 and downstream-target SCD1.	[51-52]
		Phytochemical rich fractions (free and bound phenolics)	Breast cancer	MCF-7 and MDA-MB-468	Induced the cell cycle arrest in the G2/M phase; increased the DNA fragmentation and a large number of cells accumulated in the Sub-G1 phase.	[46]
3	Proso millet	Vanillin (4-hydroxy-3 methoxybenzaldehyde)	Colon cancer	HT-29	Significantly inhibited the proliferation of HT-29 cells when treated with concentrations of 250 µg/ml and 1000 µg/ml for 48 h.	[49]
			Breast cancer	MCF-7	Significantly inhibited the proliferation of MCF-7 cells when treated with 250 µg/ml and 1000 µg/ml concentrations for 48 h; cell cycle arrest occurred in the G0/G1 phase and increased cellular growth inhibition in the Sub-G0 phase, in a dose-dependent manner.	[50]
4	Barnyard millet	Vanillin (4-hydroxy-3 methoxybenzaldehyde)	Colon cancer	HT-29	Moderately inhibited the proliferation of HT-29 cells when treated with concentrations of 250 µg/ml and 1000 µg/ml for 48 h.	[49]
			Breast cancer	MCF-7	Suppressed the tumor-producing cells in the MCF-7 line when treated with 250 µg/ml and 1000 µg/ml concentrations of phenolic extracts followed by a 48-h incubation period; cell cycle arrest occurred in the G0/G1 phase and increased cellular growth inhibition in the Sub-G0 phase, in a dose-dependent manner compared to normal cells.	[50]
5	Finger millet	Free and bound phenolics	Breast cancer	MCF-7, MDA-MB-231, and MDA-MB-468	Free phenolics (procyanidin B1, 3-O-methyl quercetin and epigallocatechin) exhibited better cytotoxicity against breast cancer cell lines compared to bound phenolics. Free phenolics induced fragmentation of DNA and apoptotic cell death in breast cancer cells.	[51]
6	Kodo millet	TPC, hydroxyl and peroxyl radical inhibition	Colon cancer	HT-29	Cell proliferation inhibition by millet grain phenolic extracts was time and dose dependent. At a lower (0.1 mg/ml) and higher (0.5 mg/ml) concentration, hulled kodo millet phenolic extracts showed 100% cellular proliferation inhibition activity. (3) At a higher concentration (0.5 mg/ml), the whole and dehulled kodo millet grain phenolic extracts showed a higher inhibition activity of 100% and 76%, respectively, against HT-29 cells, compared to their pearl millet counterparts.	[48]

The phenolics and phenolic acid derivatives, flavonoids and amino acids extracted from seeds of finger millet variety KMR 301 using 70% ethanol and 10% alkali revealed that free phenolic acids induced cell death in breast and colorectal cancer cells by inducing G0/G1 or G2/M arrest in a cell line-dependent fashion and increased the fragmentation of DNA leading to the accumulation of cells in the Sub-G1 phase [51-52].

Bioavailability, Bioaccessibility, and Delivery of Millet Bioactive Peptides

The bioactivity of peptides is determined by both the bioavailability and bioaccessibility of peptides [53]. The bioavailability, bioaccessibility, and delivery of millet bioactive peptides face significant difficulties due to the physiochemical and biological properties of peptides such as molecular size, charge, lipophilicity, solubility, and route of administration [54]. So far, peptide delivery has primarily relied on parental routes (injections and infusions), which have their own limitations. Other delivery routes, such as oral, transdermal, nasal, pulmonary, and buccal, are being considered as an alternative non-invasive strategy to deliver peptides [55].

Production and Processing of Millet Bioactive Peptides

Millet bioactive peptides should be extracted and used for further research to cure cancer. The production and processing of millet bioactive peptides involves several steps starting from extraction and purification of the protein from the source seeds, hydrolysis of the purified protein, and identification, purification, and characterization of peptides from the hydrolyzed protein. Generally, bioactive peptides are inactive within the sequence of parent proteins and become active once released through the hydrolysis process.

## Conclusions

Millets are an affordable and a nutritious option for people living in poor, low-income settings, who cannot afford expensive functional foods with high health benefits. Farmers should be encouraged to grow millets, and subsidies should be given by the government in order to boost their production and consumption. Millets' consumption should be encouraged by making people aware of the nutritional merits and overall health benefits of millets, including their potential to lower the risk of cancer in humans. The compounds found in millets that have anti-cancerous properties can be extracted and recommended for cancer cure. Anti-cancer properties of the millets should be studied further to successfully exploit the potential of these grains for the treatment of future cancer patients.
